# OPN Promotes Cell Proliferation and Invasion through NF-*κ*B in Human Esophageal Squamous Cell Carcinoma

**DOI:** 10.1155/2022/3154827

**Published:** 2022-12-15

**Authors:** Bolin Chen, Shuzhi Liang, Haibin Guo, Li Xu, Jia Li, Jie Peng

**Affiliations:** ^1^The Second Department of Thoracic Oncology, Hunan Cancer Hospital/the Affiliated Cancer Hospital of Xiangya School of Medicine, Central South University, Changsha 410013, Hunan, China; ^2^Department of Hematology Oncology, Xinning County People's Hospital, Shaoyang 422700, Hunan, China; ^3^Department of Gastroenterology, Xiangya Hospital, Central South University, Changsha 410008, Hunan, China

## Abstract

**Background:**

Osteopontin (OPN) is a phosphorylated glycoprotein. There is increasing evidence that the OPN gene played a major role in the progression of solid organ tumors. However, few studies have clarified how OPN regulated the functional role of human esophageal squamous cell carcinoma (ESCC). This study was designed to investigate the effect of OPN in esophageal squamous cell carcinoma.

**Methods:**

First, we screened Eca-109 and KYSE-510 cells to construct OPN silencing and overexpression models. Endogenous OPN of Eca-109 and KYSE-510 were knocked down or overexpressed using small interfering RNAs. QRT-PCR, Western blot, flow cytometry, and CCK-8 were used to detect the function of Eca-109 and KYSE-510 cells. Tumor formation in nude mice was used to measure tumor growth after OPN inhibition.

**Results:**

Eca-109 and KYSE-510 cells contain the si-OPN arrest cell cycle in the S-phase and increase apoptosis. These changes were OPN downregulation of the NF-*κ*B pathway that significantly reduced the protein levels of TNF-*α*, IL-1*β*, and p-p65. However, the activity of Eca-109 and KYSE-510 cells was enhanced in OPN overexpressing cells. Then, the in vivo tumor formation experiment in nude mice showed that the tumor volume and weight of nude mice after silencing OPN were significantly reduced.

**Conclusion:**

This study contributed to understanding the vital role of OPN in ESCC development and progression. This could be a promising molecular target for developing new ESCC diagnostic and therapeutic strategies.

## 1. Introduction

Esophageal cancer, also known as esophageal cancer, is a common digestive tract tumor, and its incidence and mortality rates vary widely from country to country. China is one of the regions with a high incidence of esophageal cancer in the world, and the incidence is mostly in the middle-aged and elderly population [1, 2]. The typical esophageal cancer symptom is progressive difficulty swallowing, first dry food, then semiliquid food, and finally water and saliva [3]. Most patients with esophageal cancer are already in the middle to the late stage when diagnosed with cancer, making the treatment process more passive. Therefore, early detection of esophageal cancer risk factors and prevention to reduce the occurrence of esophageal cancer are the key tasks in the prevention and treatment of esophageal cancer nowadays [4, 5]. Therefore, the search for effective molecular targets has been a research hotspot and difficulty in the field of esophageal cancer.

Osteopontin (OPN) is a phosphorylated glycoprotein [6]. OPN molecule is 41.5 KD and is an acidic secreted glycoprotein [7]. OPN can promote cellular chemotaxis, invasion, and metastasis. It was involved in immune response, promotes blood vessel growth, and inhibits apoptosis [8]. There is growing evidence that OPN genes play a regulatory role in a variety of solid cancerous tumors [9, 10]. Moreover, elevated OPN levels may be linked to poor patient prognosis [11, 12]. Highly phosphorylated human breast OPN promotes cell migration in human placental choriocarcinoma cell lines, whereas less phosphorylated OPN does not stimulate cell migration [13]. These past studies used purified OPN or recombinant OPN from noncancerous tissues for functional assays [14]. Although OPN expression has been extensively studied, the role and the exact mechanism of OPN in esophageal carcinogenesis remain unclear. Therefore, the role of OPN secreted by cancer cells in the behavior of cancer cells is not yet clear. Therefore, we attempted to examine the relationship between OPN and cancer cell proliferation and apoptosis.

OPN can activate nuclear factor-*κ*B (NF-*κ*B) to promote the secretion of ECM in chondrocytes [15]. NF-*κ*B can activate genes that maintain cell proliferation consisting of two subunits, p65 and p50 [16]. The NF-*κ*B pathway is primarily an inflammatory oncogenic signaling pathway that contributes to angiogenesis and proliferation and was constitutively activated in various human cancers [17, 18]. NF-*κ*B is a transcription factor that plays a significant regulatory role in cellular life processes, including immunity, stress response, adaptive immunity, and lymphoid organ formation [19, 20]. As an early transcription factor, activation of NF-*κ*B does not require newly translated proteins in regulation [21]. The activated NF-*κ*B is transported from the cytoplasm to the nucleus and then binds to specific DNA sequences to form DNA/NF-*κ*B complexes, which initiate transcription of downstream DNA sequences and synthesis of proteins, which in turn perform different biological functions [22]. Therefore, we attempted to explore the relationship between OPN and NF-*κ*B pathways in esophageal cancer cells.

In this study, we examined the expression of OPN in esophageal squamous carcinoma cell lines. Small interfering RNA (siRNA) knocked down the expression of endogenous OPN in esophageal squamous carcinoma cell lines. A silent OPN nude mouse tumorigenic model was constructed to elucidate the role of OPN in regulating ESCC proliferation *in vitro* and *in vivo*. The aim is to improve the understanding of esophageal cancer treatment.

## 2. Materials and Methods

### 2.1. Cell Culture

ESCC cell lines Eca-109, KYSE-510, KYSE-450, and normal esophageal cell line HEEC were purchased from BeNa Biotechnology Co., Ltd. All cells were cultured in RPMI-1640 (PM150110, Procell) medium with 10% fetal bovine serum (FBS) (Biology Industries). Cells were maintained at 37°C in a humidified air atmosphere of 5% CO_2_. Cells were divided into groups: si-NC group (Eca-109 and KYSE-510 cells were transfected with NC plasmid). si-OPN group (Eca-109 and KYSE-510 cells were transfected with OPN overexpression plasmid). The plasmid was transiently transfected into Eca-109 and KYSE-510 cells. All plasmids were obtained commercially from Abiowell (Changsha, China).

### 2.2. qRT-PCR

Total RNA is extracted from cells, and the mRNA is used as a template for reverse transcription into cDNA using Oligo (dT) or random primers. The following reaction conditions were used: predenaturation at 95°C for 10 min, 40 cycles of denaturation at 94°C for 15 s, and annealing at 60°C for 30 s. *β*-Actin was used as an internal reference primer. OPN Sense 5′-AGCAGAATCTCCTAGCCCCA-3′, Antisense 5′- ACGGCTGT CCCAATCAGAAG-3′, Sense 5′-AATCCCATCACCATCTTCCA-3′ and antisense 5′- CTTCTCCATGGTGGTGAAGA -3′ for *β*-actin. The relative quantitative method (^2^−^△△Ct^ method) was used to calculate the relative transcription level of the target gene: △△Ct = △ experimental group−△ control group, △Ct = Ct (target gene)−Ct (*β*-actin).

### 2.3. Western Blot

RIP lysate was purchased from Hanheng Bio for protein extraction. Hanheng 5X sample buffer was mixed with protein sample 4 : 1. Then, vortex oscillation was mixed. The protein samples were placed in a water bath at 95°C for 10 min. Protein PAGE gel was configured for protein gel electrophoresis. The gel glass plate after electrophoresis was removed. The following antibodies were used for gel excision of the region of the protein of interest based on Western Marker Western blotting: anti-OPN antibody (Proteintech, 22952-1-AP), anti-p65 antibody (Proteintech, 66535-1-Ig), anti-p-p65 antibody (abcam, ab76302), TNF-*α* (Proteintech, 17590-1-AP), and IL-1*β* (Proteintech, 1 6806-1-AP). This was followed by exposure to horseradish peroxidase-conjugated goat anti-mouse IgG (1 : 5000, sa00001-1, Proteintech). The membrane was immersed in Supernal Plus (k-12045-d50, Advansta, USA) for luminescence development. *β*-Actin was used as an internal reference. Protein bands were scanned using Scion image software.

### 2.4. MTT Analysis

10 *μ*L of 12 mmol/L MTT (Honorgene, AWC0118b) stock solution was added to each well. Another negative control was made by adding 10 *μ*L of MTT stock solution (AWC0118a, Abiowell) to 100 *μ*L of medium alone. The plates were incubated at 37°C for 4 h. When the cell density was higher (more than 1 × 10^5^ cells per well), the incubation time could be shortened to 2 h. 50 *μ*LL of DMSO (AWC0147a, Abiowell) was added to each well and mixed thoroughly with a pipette. The culture plate was incubated at 37°C for 10 min. The absorbance (OD) value at 490 nm was analyzed with the enzyme labeling instrument (MB-530, Huisong).

### 2.5. Flow Cytometry Analysis

Cells were collected by digestion with EDTA-free trypsin. Cells were washed twice with PBS (AWC0409, Abiowell) and centrifuged at 2000 rpm for 5 minutes to collect approximately 3.2 × 10^5^ cells. 500 *μ*L of Binding buffer was in addition to suspend the cells. After adding 5 *μ*L of Annexin V-APC, 5 *μ*L of Propidium Iodide (Solarbio, CA1020) was added, mixed at room temperature, protected from light, and the reaction was performed for 10 min. Within 1 h, and observed and detected by flow cytometry.

Cells were digested and the cell suspension was plated into a Petri dish containing a coverslip. Cells were grown on coverslips for 48 hours in a CO_2_ incubator. Take out the coverslip and operate in the following order: rinse with PBS for 3 minutes, fix in methanol: glacial acetic acid = 3 : 1 fixative for 30 minutes, then stain with Giemsa solution for 10 minutes, and finally, rinse with tap water. After drying, the coverslips were flipped on the glass slides and examined microscopically (DSZ2000X, Cnmicro).

### 2.6. Nude Mice

4-week-old BALB/c nude mice (*n* = 4 per group, Hunan SJA Laboratory Animal Co., Ltd., Changsha, China). Briefly, Eca-109 and KYSE-510 cells (5 × 10^6^) were injected into mice under the armpit. Eca-109 and KYSE-510 cells were previously transfected with NC plasmids or OPN suppressor plasmids. The experiment was completed in 40 days. Tumor volume was measured every five days. Tumor volume = width^2^ × length × 0.5. After 40 days, the tumor was removed and weighed.

### 2.7. Statistical Analysis

All data were analyzed using GraphPad Prism 9.0 software (GraphPad Software, La Jolla, CA, USA). The results were expressed as mean ± standard deviation (SD). An unpaired *t*-test was used to compare the two groups with a normal distribution. Comparisons among multiple groups were conducted using a one-way analysis of variance (ANOVA), followed by Tukey's post hoc test. Differences were considered statistically significant at *P* < 0.05.

## 3. Results

### 3.1. The Expression of OPN in the Esophageal Carcinoma and Normal Esophageal Epithelial Cell Lines

**Figure 1:**
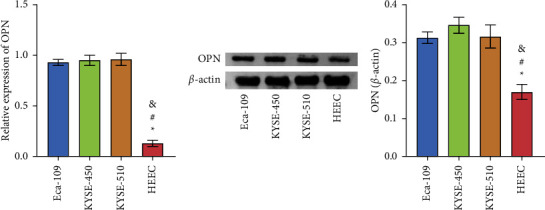
Expression of OPN in the Eca-109, KYSE-450, KYSE-510, and HEEC. (a) The mRNA of OPN was detected by qRT-PCR. (b) The protein of OPN was measured by western blot. ^*∗*^*P* < 0.05 compared with Eca-109, ^#^*P* < 0.05 compared with KYSE-450, ^&^*P* < 0.05 compared with KYSE-510.

To observe the expression of OPN in esophageal cancer cell lines, we selected Eca-109, KYSE-450, KYSE-510, and normal esophageal epithelial cell line HEEC for experiments. QRT-PCR and Western blot were used to detect the expression of OPN. The data results showed that the mRNA and protein of OPN are highly expressed in the three esophageal cancer cells (Figures 1(a) and 1(b)), and there is a statistical significance (*P* < 0.05). We selected Eca-109 and KYSE-510 cells for subsequent experiments.

### 3.2. OPN Silenced Cell Model was Constructed

**Figure 2:**
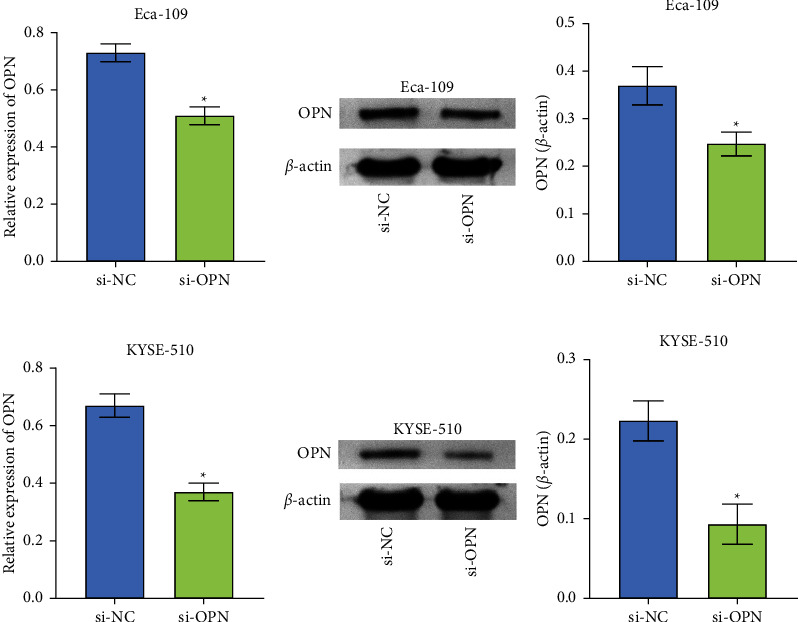
The expression level of OPN was suppressed. (a) OPN mRNA levels in Eca-109 cells after OPN silencing. (b) The level of OPN protein of Eca-109 cells in si-NC and si-OPN groups. (c) The expression of OPN mRNA in KYSE-510 cells decreased after OPN silencing. (d) The level of OPN protein in KYSE-510 after OPN silencing. ^*∗*^*P* < 0.05 compared with si-NC.

The above results showed that OPN was highly expressed in Eca-109, KYSE-450, and KYSE-510 cell lines, and there was no significant difference. We randomly selected Eca-109 and KYSE-510 cell lines for subsequent experiments. To investigate the effect of OPN on Eca-109 and KYSE-510 cells, we constructed a silent OPN cell model to inhibit the expression of OPN. As displayed in Figures 2(a)–2(d), compared to the si-NC, cells transfected with si-OPN had significantly decreased OPN mRNA or protein levels. As shown in Figure S1, OPN mRNA or protein levels were significantly higher in Eca-109 and KYSE-510 cells transfected with oe-OPN compared to oe-NC. The above data indicated that the cell model was successfully constructed.

### 3.3. The Impact of Knockdown OPN on the Proliferation of Esophageal Squamous Carcinoma Cells

**Figure 3:**
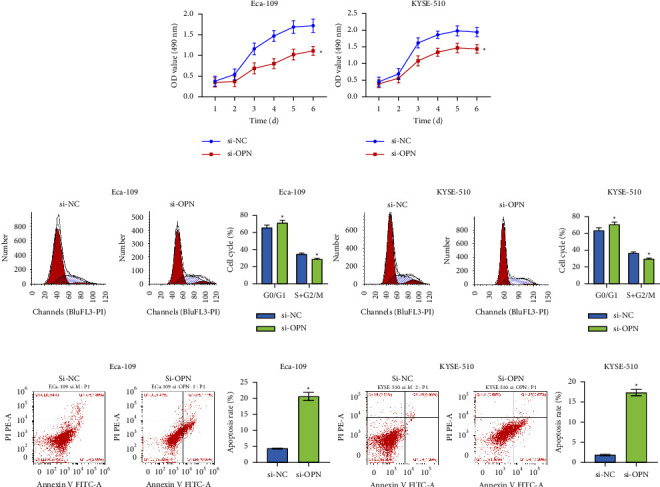
OPN knockdown could inhibit esophageal squamous carcinoma cell proliferation. (a) The proliferation ability of Eca-109 and KYSE-510 cells. (b) Cell cycle distribution of Eca-109 and KYSE-510 cells. (c) Cell apoptosis distribution of Eca-109 and KYSE-510 cells. ^*∗*^*P* < 0.05 compared with si-NC.

To study the effect of OPN silencing on the proliferation of esophageal cancer cells, the proliferation efficiency of Eca-109 and KYSE-510 cells was observed by MTT assay. Compared with the si-NC group, the proliferation ability of Eca-109 and KYSE-510 cells in the si-OPN group was considerably decreased (Figure 3(a)). Cell proliferation and cell cycle were detected by flow cytometry. The data showed that Eca-109 and KYSE-510 cells containing si-OPN blocked the S-phase cell cycle and enhanced apoptosis (Figures 3(b) and 3(c)). The result of overexpression of OPN was the opposite (Figure S2). In conclusion, silencing OPN effectively inhibited the malignant progression of cells.

### 3.4. OPN Affected the Expression of NF-*k*B in Esophageal Squamous Carcinoma Cells

**Figure 4:**
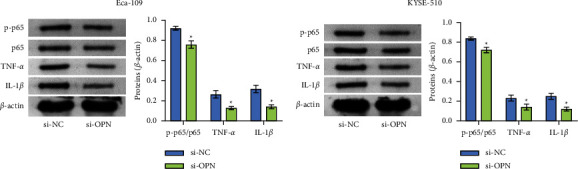
OPN could affect the level of p65 in the Eca-109 and KYSE-510 cells. (a and b) The level of p65, TNF-*α*, IL-1*β,* and p-p65 in the cells were lower in the si-OPN compared with the si-NC group. ^*∗*^*P* < 0.05 compared with si-NC.

In view of the effect of the NF-*k*B pathway on cell proliferation, we investigated whether inhibition or overexpression of OPN affected the expression of the NF-*k*B pathway in Eca-109 and KYSE-510 cells. The total cellular levels of p65 and p-p65 were analyzed by Western blot. The data showed that downregulation of endogenous OPN reduced TNF-*α*, IL-1*β,* and p-p65 protein levels (Figure 4). Up-regulation of endogenous OPN can promote the protein expression of TNF-*α*, IL-1*β,* and p-p65 (Figure S3). In conclusion, OPN had an effect on the NF-*κ*B pathway.

### 3.5. OPN Knockdown Could Inhibit Tumor Proliferation In Vivo

**Figure 5:**
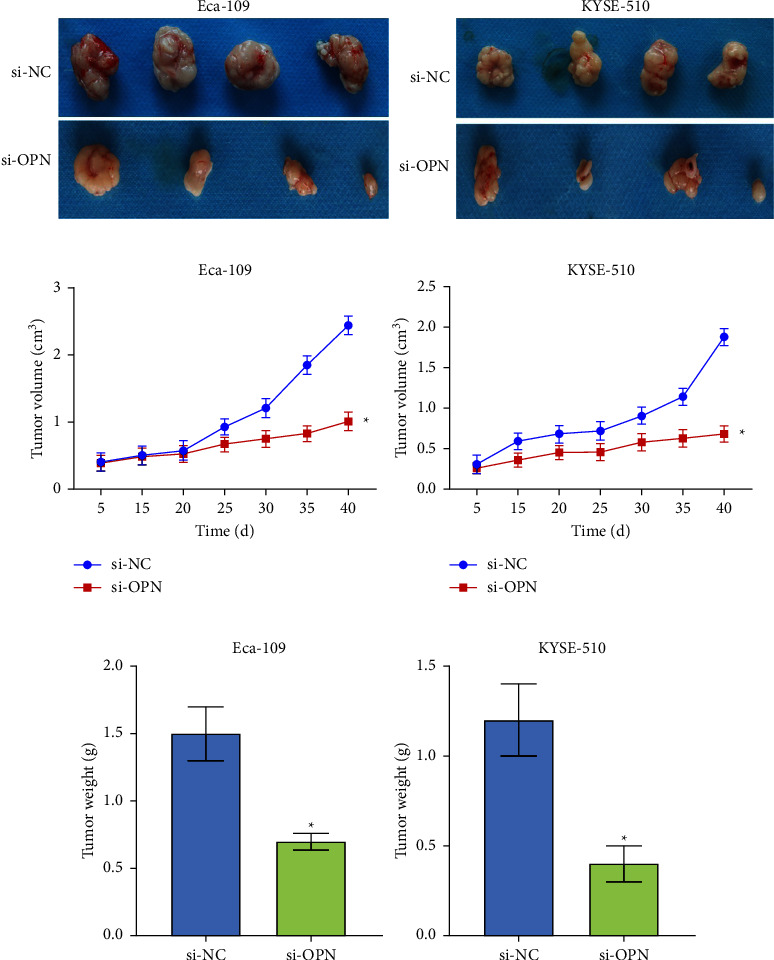
Silencing OPN can inhibit tumor growth. (a) Tumor images of si-NC and si-OPN groups. (b) Tumor volume decreased after OPN silencing. (c) Tumor weight decreased after OPN silencing. ^*∗*^*P* < 0.05 compared with si-NC. *n* = 4.

The above results indicated that inhibition of OPN *in vitro* could affect the proliferation of cells. We continued to explore whether the downregulation of OPN could inhibit tumor proliferation in vivo. Cells were transfected with OPN silencing plasmids and injected into nude mice under the armpit. Figures 5(a) and 5(b) displayed tumor growth in nude mice within 40 days. Compared with the si-NC group, the tumors in the si-OPN group appeared later and grew slowly. OPN knockout tumors were found to have significantly lighter final weight than the Control (Figure 5(c)). Collectively, these data demonstrate that OPN could encourage the development of tumor growth in vivo.

## 4. Discussion

Esophageal cancer is one of the most common GI tumors with a high mortality rate in EC patients. This article explored that silencing OPN inhibits the proliferation of Eca-109 and KYSE-510 cells. The NF-*κ*B pathway was inhibited and apoptosis was increased in Eca-109 and KYSE-510 cells with down-regulated OPN gene expression. This showed that modulation of OPN can mediate NF-*κ*B to inhibit the progression of esophageal cancer cells.

It is pointed out that the level of OPN is related to the tumor grade and prognosis of patients with bladder cancer, breast cancer, prostate cancer, and colon cancer [23–25]. This suggests that this protein was involved in tumor formation and was closely associated with tumor progression. High OPN levels are associated with lymph node metastasis. Patients with high OPN levels have smaller overall survival than those with low OPN levels [26]. In the present study, we found that OPN plays a major role in regulating ESCC cell proliferation and NF-*κ*B p65 expression. Previous studies have shown that OPN was highly expressed in ovarian cancer tissues, and overexpression of OPN promotes ovarian cancer cell proliferation and is an unfavorable factor for the survival and prognosis of ovarian cancer patients.

Targeting OPN has potential implications for providing new therapeutic opportunities for ovarian cancer patients [27]. This study used Eca-109 and SKYE-510 cells as ESCC tumor cell models and HEEC cells as normal esophageal epithelial cell models. Thus, our results agree with previous observations that the OPN gene was highly expressed in multiple cancers. In the present study, we performed siRNA-mediated OPN knockdown in a high OPN-expressing ESCC cell line. In contrast, in the present study, we performed siRNA-mediated knockdown of OPN in a high OPN-expressing ESCC cell line to determine how OPN regulates the growth of esophageal cancer cells. The results indicated that OPN had specific effects on ESCC cell proliferation. Knockdown of OPN induces reduced proliferation of ESCC cells.

It has been noted that the mechanism by which OPN enhances tumor aggressiveness may be related to nuclear factor kappa B (NF-*к*B) activation [28]. NF-*κ*B activation was associated with several tumorigenic processes [29, 30]. The NF-*к*B pathway was an important pro-inflammatory signaling pathway that plays an important role in carcinogenesis. OPN activates the NF-*к*B signaling pathway mainly by binding to integrin *α*v*β*3, ultimately leading to tumor progression [31, 32]. OPN promotes tumor growth in breast cancer by activating the CD44/NF-kappa B pathway in cells with low integrin *β*3 levels [33]. Interestingly, the knockdown of endogenous OPN reduced the proliferation of Eca-109 and KYSE-510 cells. These changes suggest that OPN downregulation significantly reduces NF-*к*B p65 and p-p65 protein levels. These results indicated that OPN could mediate the proliferation and apoptosis of Eca-109 and KYSE-510 by regulating the expression of NF-*κ*B p65. The clinical study of its in-depth mechanism needs to be further explored.

In conclusion, our data demonstrated that OPN can regulate ESCC cell proliferation through NF-*κ*B. Knockdown of OPN expression in Eca-109 and KYSE-510 cells inhibited cell proliferation and promoted apoptosis. Overexpression of OPN in Eca-109 and KYSE-510 cells promoted cell proliferation and inhibited apoptosis. This finding could serve as a basis for a potential target for treating esophageal cancer.

## Data Availability

The datasets used and/or analyzed during the current study are available from the corresponding author upon reasonable request.

## Supplementary Materials

Supplementary Materials
*Figure S1.* Overexpressing OPN was constructed in Eca-109 and KYSE-510 cells. (A) The level of OPN mRNA in overexpressing OPN of Eca-109 cells was higher. (B) The level of OPN protein in Eca-109 cells in each group. (C) The level of OPN mRNA in KYSE-510 cells was higher in the oe-OPN group. (D) The level of OPN protein in overexpressing OPN of KYSE-510 cells was higher. ^*∗*^*P* < 0.05 compared with oe-NC. *Figure S2*. Overexpression OPN could promote cells proliferation. (A) The proliferation capacity of Eca-109 and KYSE-510 cells increased after OPN overexpression. (B) Cell cycle distribution of Eca-109 and KYSE-510 after OPN overexpression. (C) Apoptosis of Eca-109 and KYSE-510 cells was inhibited after OPN overexpression. ^*∗*^*P* < 0.05 compared with oe-NC. *Figure S3*. Overexpression OPN could promote the expression of p65 in the Eca-109 and KYSE-510 cells. (A-B) The p65, TNF-*α*, IL-1*β,* and p-p65 in the cells were higher in oe-OPN compared with the oe-NC group. ^*∗*^*P* < 0.05 compared with oe-NC.
