# Supplementary figures and images for: OPN Promotes Cell Proliferation and Invasion through NF-κB in Human Esophageal Squamous Cell Carcinoma
Source: Genet Res (Camb). 2022 Dec 15;2022:3154827. doi: 10.1155/2022/3154827 (PMC9779994; doi:10.1155/2022/3154827)

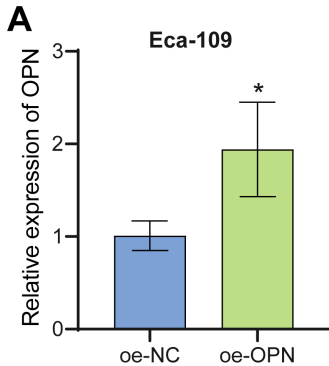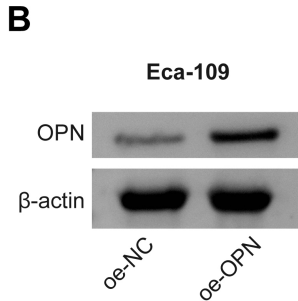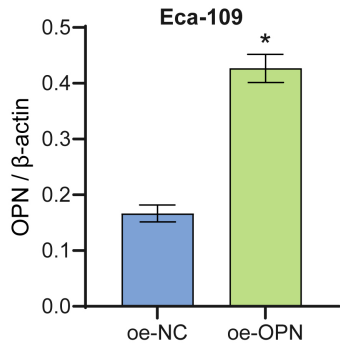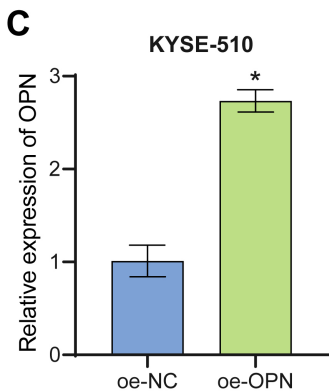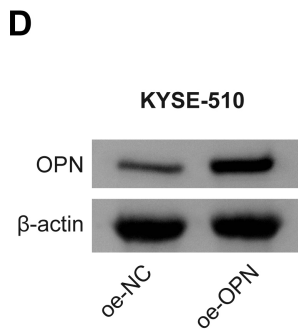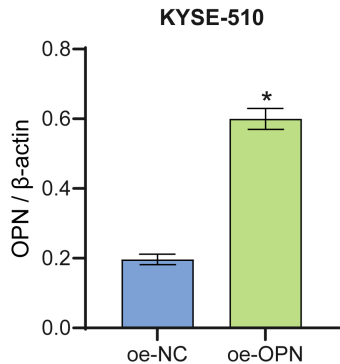

Supplement: Supplementary Materials — Figure S1. Overexpressing OPN was constructed in Eca-109 and KYSE-510 cells. (A) The level of OPN mRNA in overexpressing OPN of Eca-109 cells was higher. (B) The level of OPN protein in Eca-109 cells in each group. (C) The level of OPN mRNA in KYSE-510 cells was higher in the oe-OPN group. (D) The level of OPN protein in overexpressing OPN of KYSE-510 cells was higher. ∗P < 0.05 compared with oe-NC. Figure S2. Overexpression OPN could promote cells proliferation. (A) The proliferation capacity of Eca-109 and KYSE-510 cells increased after OPN overexpression. (B) Cell cycle distribution of Eca-109 and KYSE-510 after OPN overexpression. (C) Apoptosis of Eca-109 and KYSE-510 cells was inhibited after OPN overexpression. ∗P < 0.05 compared with oe-NC. Figure S3. Overexpression OPN could promote the expression of p65 in the Eca-109 and KYSE-510 cells. (A-B) The p65, TNF-α, IL-1β, and p-p65 in the cells were higher in oe-OPN compared with the oe-NC group. ∗P < 0.05 compared with oe-NC. [file 3154827.f1.zip › Figure S1.pdf]

**A**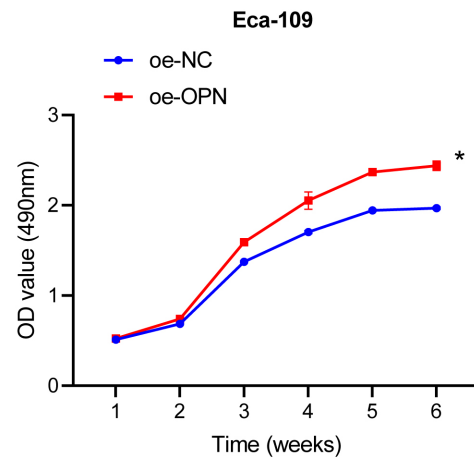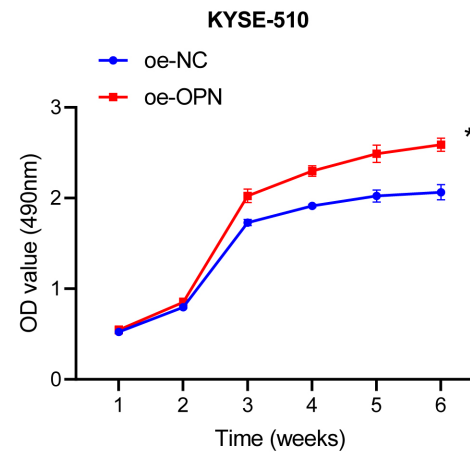**B****Eca-109**

oe-NC

oe-OPN

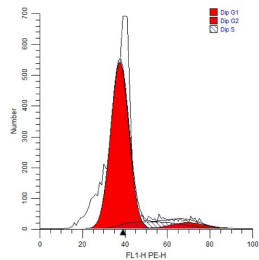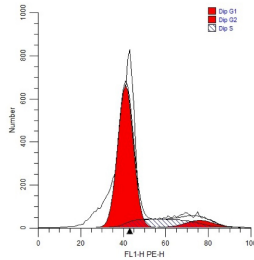**Eca-109**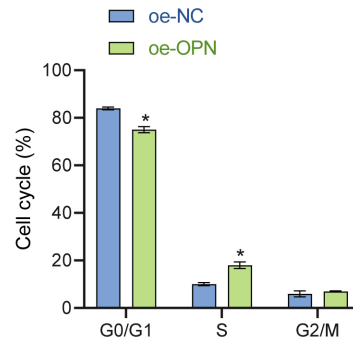**KYSE-510**

oe-NC

oe-OPN

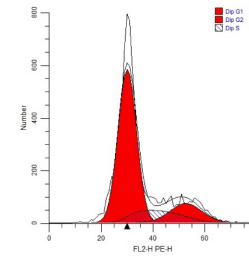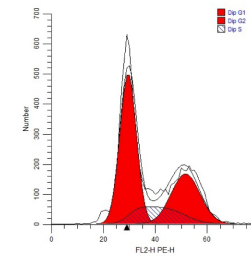**KYSE-510**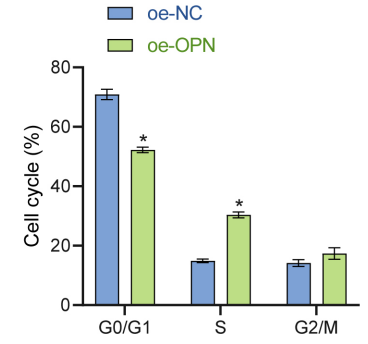**C****Eca-109**

oe-NC

oe-OPN

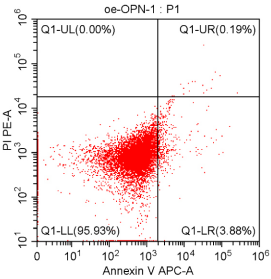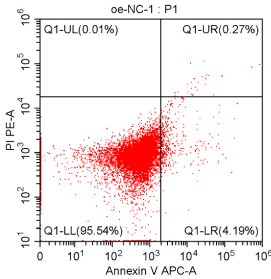**Eca-109**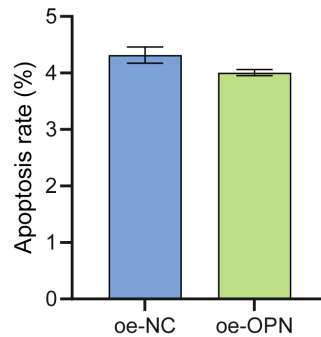**KYSE-510**

oe-NC

oe-OPN

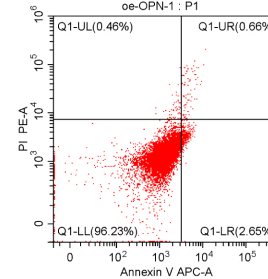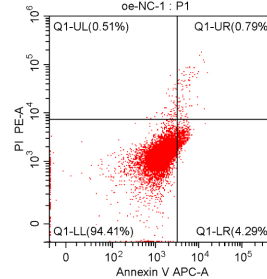**KYSE-510**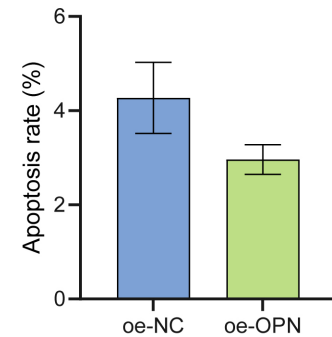

Supplement: Supplementary Materials — Figure S1. Overexpressing OPN was constructed in Eca-109 and KYSE-510 cells. (A) The level of OPN mRNA in overexpressing OPN of Eca-109 cells was higher. (B) The level of OPN protein in Eca-109 cells in each group. (C) The level of OPN mRNA in KYSE-510 cells was higher in the oe-OPN group. (D) The level of OPN protein in overexpressing OPN of KYSE-510 cells was higher. ∗P < 0.05 compared with oe-NC. Figure S2. Overexpression OPN could promote cells proliferation. (A) The proliferation capacity of Eca-109 and KYSE-510 cells increased after OPN overexpression. (B) Cell cycle distribution of Eca-109 and KYSE-510 after OPN overexpression. (C) Apoptosis of Eca-109 and KYSE-510 cells was inhibited after OPN overexpression. ∗P < 0.05 compared with oe-NC. Figure S3. Overexpression OPN could promote the expression of p65 in the Eca-109 and KYSE-510 cells. (A-B) The p65, TNF-α, IL-1β, and p-p65 in the cells were higher in oe-OPN compared with the oe-NC group. ∗P < 0.05 compared with oe-NC. [file 3154827.f1.zip › Figure S2.pdf]

**A****Eca-109**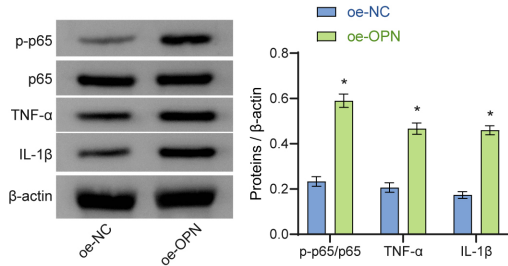**B****KYSE-510**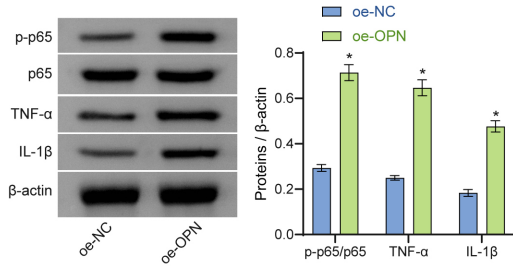

Supplement: Supplementary Materials — Figure S1. Overexpressing OPN was constructed in Eca-109 and KYSE-510 cells. (A) The level of OPN mRNA in overexpressing OPN of Eca-109 cells was higher. (B) The level of OPN protein in Eca-109 cells in each group. (C) The level of OPN mRNA in KYSE-510 cells was higher in the oe-OPN group. (D) The level of OPN protein in overexpressing OPN of KYSE-510 cells was higher. ∗P < 0.05 compared with oe-NC. Figure S2. Overexpression OPN could promote cells proliferation. (A) The proliferation capacity of Eca-109 and KYSE-510 cells increased after OPN overexpression. (B) Cell cycle distribution of Eca-109 and KYSE-510 after OPN overexpression. (C) Apoptosis of Eca-109 and KYSE-510 cells was inhibited after OPN overexpression. ∗P < 0.05 compared with oe-NC. Figure S3. Overexpression OPN could promote the expression of p65 in the Eca-109 and KYSE-510 cells. (A-B) The p65, TNF-α, IL-1β, and p-p65 in the cells were higher in oe-OPN compared with the oe-NC group. ∗P < 0.05 compared with oe-NC. [file 3154827.f1.zip › Figure S3.pdf]
